# Ultrastructural Examination of the Fungus‐To‐Fungus Interactions of *Lecanicillium uredinophilum* and *Phakopsora pachyrhizi*

**DOI:** 10.1002/pei3.70082

**Published:** 2025-09-01

**Authors:** P. P. Mwelasi, M. D. Laing, J. D. Ibaba, R. Rogers, M. Z. Németh, K. S. Yobo

**Affiliations:** ^1^ Discipline of Plant Pathology, College of Agriculture, Engineering & Science; School of Agricultural, Earth and Environmental Sciences University of KwaZulu‐Natal Pietermaritzburg Republic of South Africa; ^2^ Institut de Recherche Agricole et Forestière (IRAF) Centre National de la Recherche Scientifique et Technologique Libreville Gabon; ^3^ HUN‐REN Centre for Agricultural Research Martonvásár Hungary; ^4^ Department of Plant Anatomy Institute of Biology, Eötvös Loránd University Budapest Hungary

**Keywords:** biotrophic, *Glycine max*, hyperparasite, *Lecanicillium uredinophilum*, mycoparasitism, *Phakopsora pachyrhizi*

## Abstract

Asian soybean rust (ASR) is caused by the biotrophic fungus *Phakopsora pachyrhizi* Syd. & P. Syd., and is one of the most important diseases of soybean [ *Glycine max* (L.) Merr.], with recorded yield losses of up to 100%. The fungus *Lecanicillium uredinophilum* isolate PP2018‐001, originally isolated from wild strawberry rust pustules, revealed hyperparasitic abilities on the rust urediniospores of soybean and frangipani (*Plumeria* spp.). This study examined the fungus‐to‐fungus interactions of the hyperparasite (*L. uredinophilum*) and *P. pachyrhizi* using confocal laser‐scanning microscopy (CLSM), transmission electron microscopy (TEM) and scanning electron microscopy (SEM) to reveal whether *L. uredinophilum* isolate PP2018‐001 employed mycoparasitism as its mechanism of action. The CLSM used an *Agrobacterium tumefaciens‐*mediated transformation (AGTM) of *L. uredinophilum* isolate PP2018‐001 with the green fluorescence protein (GFP) gene to track the hyperparasite infection process. The SEM and TEM investigations used both in vivo and in vitro co‐inoculations to examine the extent and type of damage caused by *L. uredinophilum* on the urediniospores of *P. pachyrhizi*. Confocal microscopy revealed the ability of *L. uredinophilum* to penetrate and intensely colonize urediniospores within 36 h. In SEM studies, *L. uredinophilum* hyphae extensively coiled around urediniospores after both the in vivo and in vitro co‐inoculations, with clear penetration and damage of outer urediniospore walls, which, with time, produced visible perforations and loss of cell integrity. TEM revealed the infection of urediniospores by *L. uredinophilum,* penetrating hyphae, germ tube pores, and the collapse of urediniospores. This study captured the parasitic nature of *L. uredinophilum* on *P. pachyrhizi* fungus‐fungus interactions. It showed that mycoparasitism occurred, and possibly enzymatic activity occurred, resulting in the degradation of urediniospore outer walls and germ tubes. The results are important for the potential registration of isolates of *L. uredinophilum* for the biological control of soybean rust.

## Graphical abstract

*Lecanicillium uredinophilum* colonizes and penetrates the soybean rust pathogen (*Phakopsora pachyrhizi*) urediniospores using them as a nutritional substrate through mycoparasitism.

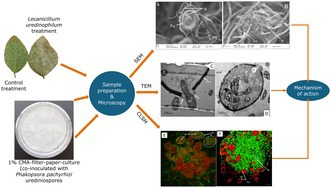

## 1 Introduction

Soybean [ *Glycine max* (L.) Merr.] is a global crop that contributes substantially to food security, provides essential nutrients, and is a significant source of protein and oil (Dourado et al. 2011; Hamza et al. 2024). Its cultivation is critical for food security and economic stability in many countries (Nair et al. 2023). The total world soybean production in 2023 was 378 million tons, with the top three world producers being Brazil (162 million tons), US (116 million tons) and Argentina (25 million tons) (Soystats 2024). South Africa was the largest producer (2.8 million tons) of the African total of 7.3 million tons (FAO 2024). However, the profitability of soybean production is negatively impacted by the obligate biotrophic fungus *Phakopsora pachyrhizi* Syd. & P. Syd., the causative agent of Asian soybean rust (ASR). The ASR pathogen results in significant yield losses (up to 100%) and financial repercussions for farmers and the agricultural industry (Hossain et al. 2024; Meira et al. 2020). In sub‐Saharan Africa (SSA), losses due to the ASR impacts have been estimated at over US$ 0.06 billion per year (Sileshi and Gebeyehu 2021).

*P. pachyrhizi* is a basidiomycete with a broad host range of at least 150 species across 53 genera of legumes. It has an interesting life cycle which has been reviewed by Goellner et al. (2010). The ASR pathogen produces urediniospores which are known to be infective, and teliospores postulated as resting propagules not known to infect or germinate naturally (Goellner et al. 2010). However, teliospores have been induced under laboratory conditions to produce basidiospores (Yeh et al. 1981). A urediniospore landing on a soybean leaf surface would require leaf wetness of 6 to 12 h, temperatures between 15°C and 28°C, and RH (> 75%) for successful germination (Goellner et al. 2010) and appressorial formation, which establishes the host‐pathogen interactions, typical of biotrophic fungi (Mapuranga, Zhang, Zhang, and Yang 2022; Mapuranga, Zhang, Zhang, Chang, and Yang 2022). Uredinia are formed between 5 and 8 days post‐infection and would subsequently produce urediniospores within 8 to 10 days post‐infection (Goellner et al. 2010). A single infectious lesion can support urediniospore production for at least 15 weeks with a total urediniospore count of over 6000 in its lifespan (Kassie et al. 2023). The prolific spore production facilitates rapid, polycyclic disease progression and facilitates long distance dispersal via wind currents, leading to exodemic infections and ultimately regional ASR epidemics (Kassie et al. 2023; Santiago‐Pérez et al. 2022). Since its first report in 2001, ASR has consistently occurred across all major soybean‐producing areas in South Africa each season. However, the severity of outbreaks has varied (A. Jarvie, personal communication, May 23, 2023). The biology of *P. pachyrhizi* in its uniquely large genome (1.25 Gb), and continued studies has largely informed available management strategies (Gupta et al. 2023; Schubert 2022).

Developing disease‐resistant crops and applying chemical pesticides are the primary approaches for controlling plant diseases such as ASR (Hao et al. 2024; Langenbach et al. 2016). Fungicides in the classes of succinate dehydrogenase inhibitors (SDHIs) and quinone outside inhibitors (QoIs) effectively control ASR. However, in Brazil alone, the amount of fungicide required to control the ASR pathogen has increased since the first outbreak and, thus, raised the costs of soybean production (Meira et al. 2020). The sole use of a few classes of fungicides has led to pathogens developing resistance, restricting their application in disease management (Klosowski et al. 2018; Schubert 2022; Sharma et al. 2019; Tudi et al. 2021). Despite breeding efforts over the years to develop soybean cultivars with durable resistance to the ASR pathogen, its complex genome contributes to its genetic variability and adaptability, resulting in the erosion of the conferred resistance (Schubert 2022). To date, there are no soybean cultivars that offer long‐term resistance to the ASR pathogen (Chicowski et al. 2024).

In the quest for sustainable farming practices, biological control agents (BCAs) against the ASR pathogen have emerged as a promising alternative to chemical fungicides (Dorighello et al. 2020; Si et al. 2022; Twizeyimana et al. 2023). The BCAs can potentially manage plant diseases with reduced environmental impact and little risk of the pathogen developing resistance. Among the potential biocontrol agents, isolates of *Lecanicillium* spp. Zare & W. Gams. (syn. to *Verticillium* spp., *Akanthomyces* spp.) (Kepler et al. 2017) are effective BCAs for numerous insect pests (Manfrino et al. 2022; Zhou et al. 2020). *Lecanicillium* spp. have been evaluated and shown potential as BCAs on *Hemileia vastatrix* Berk. & Broome, the rust pathogen of coffee ( *Coffea arabica* L.) (Belachew Bekele 2022; Das et al. 2024; García‐Nevárez and Hidalgo‐Jaminson 2019; Luiz et al. 2024; Romero and Castillo‐Arévalo 2023) as well as *Puccinia arachidis* Speg., the peanut ( *Arachis hypogaea* L.) rust pathogen (Nana et al. 2023, 2024).

The hyperparasitic nature of *L. uredinophilum* was first reported by Park et al. (2015), who isolated three isolates from rust pustules during extensive surveys on hyperparasitic fungi infecting rusts in Korea. Subsequent work by Manfrino et al. (2022) expanded its known host range by isolating the species from both fungal and insect hosts and proposed its reclassification as *Akanthomyces uredinophilus* comb. nov. However, in this study, the original designation *L. uredinophilum* was retained to maintain consistency with the prevailing usage and due to ongoing taxonomic uncertainty within the genus. Over the past decade, *Lecanicillium* has undergone extensive phylogenetic reassessment, with many species transferred to *Akanthomyces* (Aini et al. 2020; Kepler et al. 2017). In addition, the description of additional *Lecanicillium*‐like species within *Akanthomyces* Chen et al. (2020) further highlights the polyphyletic nature of the *Lecanicillium* genus. Molecular clock analyses suggest that the divergence of *Lecanicillium* lineages occurred relatively within Cordycipitaceae, further complicating attribution and generic boundaries (Zhou et al. 2022). These findings advance the continuing attribution challenges, particularly for entomopathogenic and mycoparasitic taxa. Further integrative taxonomic studies, combining multilocus phylogenetics, divergence time estimation, morphology, and ecology, remain necessary to resolve species boundaries within this complex genus.

The present study's central question was whether the fungus *L. uredinophilum* functions as a true mycoparasite. Specifically, the study aimed at determining if the fungus directly penetrated the host tissue (*P. pachyrhizi*) to extract nutrients from the biotrophic fungus (substrate). Although studies of *L. lecanii* on peanut rust pathogen revealed a confrontation of urediniospores, including internal invasion, as well as some presence of mucilaginous matrix suggestive of enzyme activity (Nana et al. 2023), *L. uredinophilum* has not been investigated on *P. pachyrhizi*, the soybean rust pathogen. Understanding the mode of action of *L. uredinophilum* isolate PP2018‐001 is crucial for several reasons. If *L. uredinophilum* isolate PP2018‐001 is a true mycoparasite of the ASR pathogen, it may directly attack and kill the rust fungus, providing a robust biocontrol strategy. Alternatively, if the biocontrol agent primarily relies on enzymatic activity, then insights into these mechanisms can inform the development of formulations designed to enhance such enzymatic functions, thereby improving its overall efficacy. Previous studies found that *Lecanicillium* spp. employed mechanical force and enzymes to penetrate their insect hosts (Goettel et al. 2008; Hajji‐Hedfi et al. 2023).

This study sought to elucidate the parasitic nature of *L. uredinophilum* isolate PP2018‐001 through detailed microscopic investigations. Confocal laser‐scanning microscopy (CLSM) used a green fluorescent protein (GFP) transformant to visualize *L. uredinophilum* and the interaction thereof with *P. pachyrhizi* urediniospores, whereas scanning electron microscopy (SEM) elucidated the interactions of *L. uredinophilum* and *P. pachyrhizi* in in vivo and in vitro assays. The SEM revealed direct penetration or infection of *P. pachyrhizi* by *L. uredinophilum*. Internal interactions of the hyperparasite with the ASR pathogen at the cellular level, in in vivo and in vitro assays, were visualized with transmission electron microscopy (TEM). These insights advance the understanding of fungal biocontrol mechanisms and contribute to developing more effective and sustainable integrated pest management strategies for soybean cultivation.

## 2 Materials and Methods

### 2.1 Soybean Host Plants Preparation

Seeds of an unnamed soybean cultivar susceptible to the ASR were provided by Pannar Seed Company (Corteva), Greytown, KwaZulu‐Natal (GPS coordinates: −29.058956, 30.592695). Four to five soybean seeds were planted in composted pine bark growth media (3 kg) mixed with 15 g of controlled release fertilizer (Osmocote Exact Mini 5‐6M 15‐3.9‐9.1+1.2Mg+TE, Greenhouse Products (Pty) Ltd., South Africa) in 30 cm plastic containers and subsequently moved from the media preparation room to a passive glasshouse facility (University KwaZulu‐Natal controlled environmental facilities). Osmocote is a slow‐release water‐soluble fertilizer that can provide balanced fertilization for 3–6 months, depending on the application rate and the crop. Plants were irrigated every third day using a manually controlled drip irrigation system (1 h each time). Four planting dates with an interval of 7 to 10 days were scheduled to allow for different ages of the soybean host plants, whilst assessing the inoculum of *P. pachyrhizi*. The initial *P. pachyrhizi* inoculum was obtained from the Ukulinga Research Farm, University of KwaZulu‐Natal (GPS: 29° 40′ S; 30° 24′ E; 809 m a.s.l). ASR‐infected leaves were pinned on trifoliates of soybean plants in pots to allow for natural infection. Fourteen days post‐inoculation, most potted plants (> 80%) had ASR symptoms. The infections were allowed to further proliferate for at least 4 to 6 weeks before urediniospore collection. A total of five pots, each with four (4) soybean plants, were kept in a separate glasshouse establishment to prevent them from being infected by the soybean rust pathogen.

### 2.2 Use of Frangipani Rust (*Coleosporium plumeriae*) as a Host to Maintain the Pathogenicity of Isolate PP2018‐001 of *L. uredinophilum*

In the grounds of the greenhouse facility of the University of KwaZulu‐Natal, Pietermaritzburg campus, is a large frangipani tree (*Plumeria* spp.), approximately 40 years old. Annually, the leaves are infected by the frangipani rust (*Coleosporium plumeriae* Pat.) from late October to late June in the subsequent year, summer to autumn. *Coleosporium plumeriae* provides an excellent experimental model to test potential soybean rust mycoparasites. Over the years of this study, 15 to 20 potted trees were grown from cuttings to be used in glasshouses. A conidial suspension of isolate PP2018‐001 of *L. uredinophilum* was inoculated onto the *C. plumeriae* sori twice annually and re‐isolated as a process to retain the pathogenicity of the biocontrol agent on rusts. This was done to curtail the loss of virulence, probably through epigenetics, which can occur when cultures are continuously sub‐cultured on artificial growth media (Hussien et al. 2021; Ibrahim et al. 2002; Morrow et al. 1989).

### 2.3 Soybean Rust Spore Collection and Germination Tests

Soybean plants previously infected with ASR provided the required urediniospores. These were collected 48 h before each experiment (CLSM and TEM). They were collected with a soft paintbrush and dusted into a weighing boat before being transferred into 20 mL scintillation vials and stored at 4°C ± 2°C until required for extended storage periods. The urediniospores were dried in a desiccator for 24 h and stored at −80°C. As recommended (Bonde et al. 2006), the urediniospores previously stored at −80°C were carefully rehydrated by transferring 1 mg onto a 150‐mm glass petri dish lid placed inside a glass desiccator with a film of water underneath the petri dish, and left in situ for 24 h. Germination tests were run to confirm the viability of the urediniospores before being used for the in vitro assay sample preparation required for visualization in the CLSM. The germination tests were carried out by dusting approximately 1 mg of urediniospores onto 1.25% water agar plates and incubating at 25°C ± 1°C for 24 h in the dark using a method from Gauthier et al. (2014). As recommended (Gauthier et al. 2014), the urediniospores were considered successfully germinated if the germ tube length was at least the size of the urediniospore, and samples with an 80 to 100% germination rate were used in subsequent experiments. Germinated urediniospores were viewed under a light microscope (Carl Zeiss light microscope, Model GmbH 37081, Gottingen, Germany) at ×400 resolution.

### 2.4 *Lecanicillium uredinophilum* Preparation

The biological control agent used in this study was isolate PP2018‐001, previously identified as *L. uredinophilum* based on multilocus phylogenetic analysis. Molecular characterization was conducted using the ITS, RPB2, and TEF gene regions, with the corresponding GenBank accession numbers PP259072.1 (ITS), PP273269.1 (RPB2), and PP273271.1 (TEF) deposited in the NCBI GenBank (https://www.ncbi.nlm.nih.gov/genbank/) in previous work. Isolate PP2018‐001 was isolated from colonized pustules of the wild strawberry rust, *Phragmidium mucronatum* (Pers.) Schltdl. Previous test experiments (data not shown here) with isolate PP2018‐001 had achieved at least 80% colonization of soybean rust pustules in vivo (greenhouse test experiment) and in detached leaf experiments on its colonization of pustules of *C. plumeriae*. These experiments confirmed that *L. uredinophilum* functions as an antagonist capable of infecting multiple rust species. Koch's postulates were fulfilled by conducting controlled assays to rule out the presence of other opportunistic or latent mycoparasites within the rust sori of both *Plumeria* spp. and soybean. The results demonstrated that only the rust pathogens were present on the infected leaves, with no evidence of colonization by any additional fungal antagonists or mycoparasites. Working cultures of *L. uredinophilum* were maintained on petri dishes of full‐strength PDA. The plates were stored at 4°C under continuous darkness. To track the biocontrol agent in vivo, isolate PP2018‐001 was transformed with GFP and used in subsequent experiments. The transformed isolate PP2018‐001 of *L. uredinophilum* shall be referred to as isolate PP2018‐001‐GFP.

### 2.5 Green Fluorescent Protein Transformation of *Lecanicillium uredinophilum*

**FIGURE 1:**
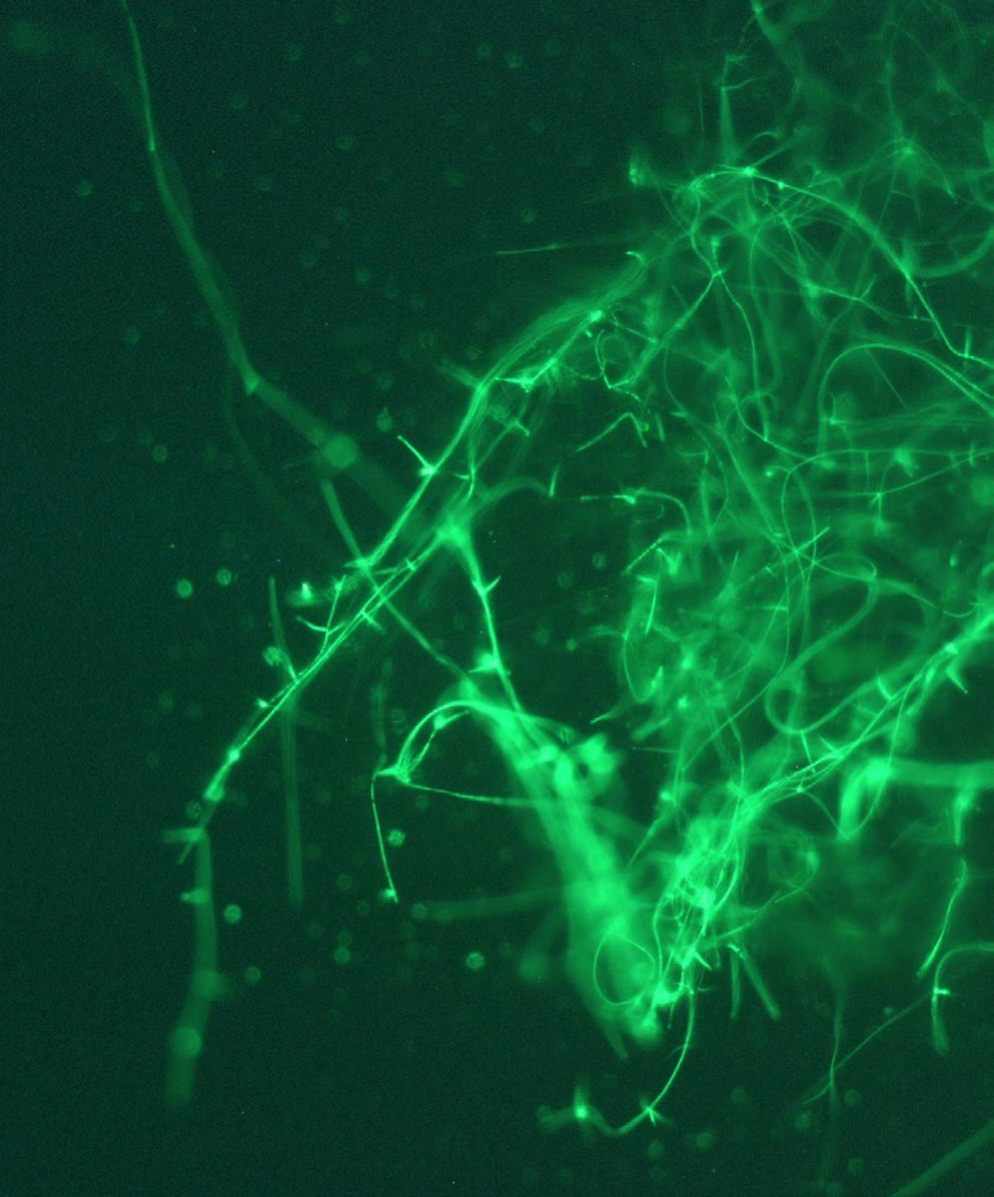
Fluorescence microscopy of a sample of *Lecanicillium uredinophilum* (PP2018‐001‐GFP), confirming its successful GFP transformation.

*Agrobacterium tumefaciens* (syn. *Rhizobium radiobacter* ) isolate AGL1 (Lazo et al. 1991) was used to transform *L. uredinophilum* (isolate PP2018‐001) with the binary vector pCBCT (Gorfer et al. 2007) that codes for the reporter gene GFP, as described by Németh et al. (2019). Fifteen to 20 small fungal colony fragments of *L. uredinophilum*, approximately 2–4 × 2–4 mm, were transferred onto sterile cellophane sheets placed on malt extract agar (MEA; Merck) plates (*d* = 9 cm). Colonies of *L. uredinophilum* (isolate PP2018‐001) were grown for 4 days in the dark at 25°C on the MEA. The cellophane sheets bearing the growing fungal colonies were then transferred to plates containing Moser induction medium (MoserIND) (Gorfer et al. 2007). *Agrobacterium tumefaciens* isolate AGL1 carrying the binary vector pCBCT was grown overnight at 28°C in 4 mL Lysogeny Broth (L.B. broth) containing 0.1% glucose supplemented with 50 μgmL^−1^ kanamycin under continuous shaking (180 rpm). Bacteria were then pelleted by centrifugation for 9 min at 3800× *g* and resuspended in *Agrobacterium* Induction Medium (AtIND) (Gauthier et al. 2014). Induction of bacteria was achieved after incubation for 6 h under continuous agitation at 180 rpm at 28°C. Aliquots (50–100 μL) of the induced bacterial culture were pipetted directly onto *L*. *uredinophilum* colonies growing on cellophane sheets placed on MoserIND and wetted by pipetting the suspension up and down. Co‐culture plates were incubated for 4 days at room temperature. The cellophane sheets from these plates were then transferred to new plates containing selective medium (MEA with 50 mgL^−1^ hygromycin B and 100 mgL^−1^ cefotaxime, Duchefa Biochemie) and incubated in the dark at 22°C for 2 to 4 weeks, until the emergence of visible fungal colonies. Hyphae of actively growing putative transformants were illuminated with blue light (400–500 nm), and colonies exhibiting green fluorescence were transferred to a new 6 cm diameter petri dish containing the same selective medium. Colonies exhibiting fluorescence when excited with blue light (400–500 nm) were considered *L. uredinophilum*‐GFP transformants. GFP expression of the subcultured colonies was verified with a fluorescence microscope (Zeiss Axioskop 2 Plus microscope, Carl Zeiss Microscopy GmbH, Germany) (Figure 1). The transformants were subcultured every 4 to 6 weeks on MEA plates. GFP expression of hyphae was verified with fluorescent microscopy before every subculturing. Hygromycin B was not added to MEA used to maintain the transformants in culture after the third transfer, as these remained stable without selection pressure during the duration of this work.

### 2.6 Scanning Electron Microscopy

#### 2.6.1 Soybean Plants Preparation and Inoculation With Soybean Rust

Colonized rust sori appear to be covered with the mycelium of the mycoparasite; however, visual examination does not reveal the nature of the microbe‐microbe interactions. Scanning electron microscopy (SEM) was employed: (i) to examine the topographical characteristics of the hyperparasitized sori of *P. pachyrhizi* and *C. plumeriae*; and (ii) to assess the integrity of the urediniospores after colonization, aiming to determine the extent of damage, if any, to the urediniospores. The SEM studies were conducted according to Gauthier et al. (2014) and Adendorff (1998), with some modifications. Modifications to the protocol included the use of potted soybean plants instead of detached leaf cuttings. These plants were naturally infected by placing rust‐infected pots adjacent to healthy ones, allowing for passive urediniospore transmission. Additionally, no spore collectors were used to harvest urediniospores due to the unavailability of such equipment in the laboratory. At the time of the successful transformation of *L. uredinophilum* with the GFP, there was a range of ASR‐infected soybean plants and non‐infected plants (growth stages V1 to R6) ready for use. The growth stages of soybean are divided into two main categories: vegetative (V) and reproductive (R) stages. Vegetative development begins with emergence (VE), followed by the appearance of unifoliate leaves (VC), and then progresses through successive trifoliate stages (V_1_ to V_*n*_), where n represents the number of fully developed trifoliate leaves, which varies depending on the cultivar and environmental conditions. The reproductive stages, designated R1 through R8, encompass the period from initial flowering to full physiological maturity (https://crops.extension.iastate.edu/encyclopedia/soybean‐growth‐stages). The R6 growth stage was defoliated due to extensive infection by *P. pachyrhizi*. Some infected plants were used to infect naturally V3 and R1 ASR‐free soybean plants to prepare the in vivo assays for the SEM studies.

#### 2.6.2 Hyperparasite Preparation and Inoculation

**FIGURE 2:**
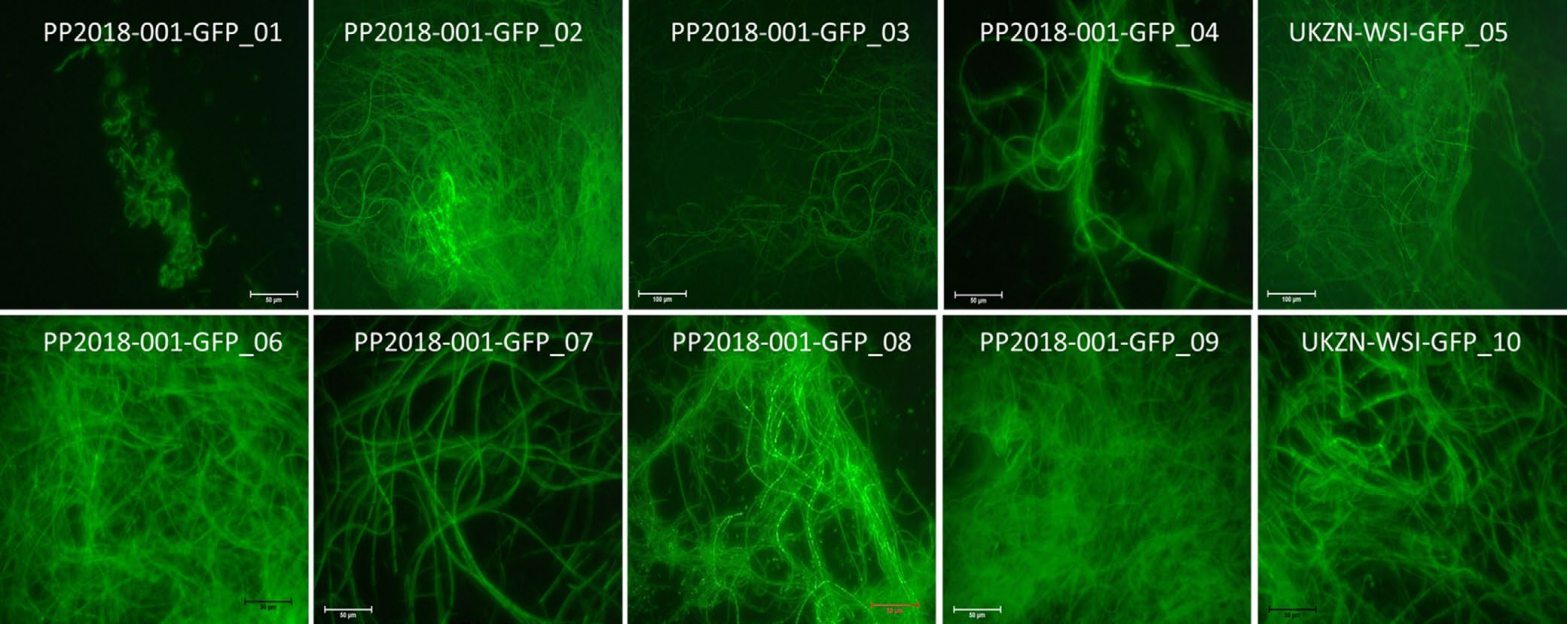
Sample images of ten transformed isolates of *Lecanicillium uredinophilum* Isolate PP2018, demonstrating the intensity of the fluorescence of the GFP.

Ten successful transformants, labeled PP2018‐001‐GFP_01 through PP2018‐001‐GFP_10 (Figure 2), were subcultured after transformation. Inoculum for transformants was prepared from subculturing on full‐strength PDA for 14 days and harvesting conidia. A 0.05% Break‐thru (Nulandis, Evonik, South Africa) solution (an adjuvant) was prepared and used to wash off conidia from plates of the ten PP2018‐001‐GFP transformants. The suspension was filtered through a double layer of cheesecloth to create conidial suspensions for the ten PP2018‐001‐GFP transformants. The concentrations of the conidial suspensions were adjusted using an improved Neubauer hemocytometer (model 3048‐13, Hausser Scientific, Horsham, PA, USA) to achieve 1 × 10^6^ conidia mL^−1^ in the in vivo assays. After 28 days of soybean rust growth on previously uninfected V3 and V1 soybean plants, three plants with leaves varying in rust disease severity (18%, 45%, and 80%) were sprayed to runoff using a pneumatic spray bottle. The plants were allowed to dry in the shade for 2 h at ambient temperature (approx. 26°C), then covered with plastic bags and transferred to the glasshouse to enable colonization of the rust pustules. After conidia of *L. uredinophilum* were sprayed onto detached rust‐infected leaves of frangipani plants, the leaves were placed in new and sterile transparent polyethylene lunch boxes (25 × 15 × 10 cm) to act as humidity chambers. The boxes were incubated at 25°C ± 2°C.

#### 2.6.3 Leaf Sample Preparation for Scanning Electron Microscopy

Leaf samples for SEM and TEM were chosen based on the extent of hyperparasitic colonization of sori (assessed visually) on days 5, 10, and 15 post‐inoculation with *L. uredinophilum*. Three samples were taken for each sampling day and each host, totaling 18 SEM‐leaf samples. Before primary fixation for SEM imaging, soybean leaf cuttings and frangipani leaf cuttings with visible colonization by *L. uredinophilum* were viewed under a Leica stereomicroscope fitted with a Leica 450FC camera, equipped with Leica LAS imaging software (Leica MZ16 Stereo Light Microscope, Germany). From the colonized portions of the leaf samples, about 8 to 10 samples of 4 mm^2^ were cut and fixed (primary fixation) in 3% buffered glutaraldehyde in 0.05 M sodium cacodylate buffer, allowed to stand for 1 h, before storage at 4°C for 4 weeks.

#### 2.6.4 Co‐Inoculations on Cornmeal Agar Filter Paper Culture

The PP2018‐001‐GFP transformants of *L. uredinophilum* were grown on 1% cornmeal agar (CMA) topped with Whatman #10 filter paper before the agar had set (CMA‐filter‐paper‐culture). After the media had set, 1000 μL of PP2018‐001‐GFP conidial suspension (1 × 10^6^ spores mL^−1^) was pipetted onto the plate surface and spread with a sterile L‐shaped glass rod. The plates were incubated at 25°C ± 2°C for 14 days. On day 15, urediniospores (1 mg) previously stored for 48 h (germination of 80%–100%) were dusted onto the 14‐day CMA‐filter‐paper‐culture with the aid of a paintbrush and incubated at 25°C ± 2°C for 5 days. Four segments of 4 mm^2^ of the CMA‐filter‐paper‐culture, post‐inoculation with urediniospores, were cut every 24–36 h using a sharp razor blade and submerged in 3% glutaraldehyde in 0.05 M sodium cacodylate buffer, held at room temperature for 1 h before refrigeration at 4°C. After the primary fixation of both leaf (Section 2.6.3 above) and CMA‐filter‐paper samples, 2 × 5 min of buffer washes were made in 0.05 M sodium cacodylate buffer. The samples were rinsed in 0.05 M sodium cacodylate buffer again for the same time and rinsed twice in the sodium cacodylate buffer. This was followed by a series of dehydration steps in ethanol, starting at 10% and then incrementally to 30%, 50%, 70%, and 90%, each at 10 min and a single wash. Dehydration in 100% ethanol was done three times at 10‐min intervals. After this series of dehydration, the samples were critical point dried (Quorum Q150 RES Critical Point Dryer, Quorum Technologies Ltd., United Kingdom) using carbon dioxide as the transition fluid followed by the affixation of 2–3 samples of 4 mm^2^ to stubs, which were subsequently sputter‐coated with 60:40 gold: palladium (Quorum Q150R ES Gold Sputter Coater, Quorum Technologies Ltd., United Kingdom). The SEM samples were viewed using a Zeiss EVO LS 15 Variable Pressure Scanning Electron Microscope, VP SEM (Carl Zeiss, Oberkohen, Germany).

### 2.7 Confocal Laser‐Scanning Microscopy (CLSM)

Scanning electron microscopy (SEM) offers extensive imagery to resolve some of the critical aspects of the anatomical structures of fungi; however, it cannot visualize internal structures. The CLSM studies were done using the techniques of Gauthier et al. (2014). Optical sectioning was possible through the CLSM instrument (Zeiss LSM 710 Confocal Laser Scanning Microscope, Zeiss, Oberkochen, Germany). Conidia of the ten PP2018‐001‐GFP transformants were actively grown on full‐strength PDA and incubated at 25°C ± 2°C for 14 days. After 14 days, wet mounts from each of the isolates were viewed under a compound microscope (Olympus AX70, Olympus USA, Melville, NY, USA) to confirm green fluorescence (the microscope was equipped with a GFP filter with 450–490 nm excitation, 500 nm emission) and with a Nikon DS Fil camera for imaging. Previously, the ten PP2018‐001‐GFP transformants successfully colonized urediniospores when viewed under SEM in in vivo preparation experiments. The transformant PP2018‐001‐GFP_08 had a slightly higher GFP signal than other transformants. However, all of the ten transformants could have been used, based on in vivo SEM preparation experiments. Representative images are presented in Figure 2, which were captured using a compound light microscope equipped for fluorescence imaging (Olympus AX70, Olympus USA, Melville, NY, USA), fitted with a Nikon DS‐Fi1 camera and operated via NIS‐Elements D Imaging Software. Growth media (1% CMA) was prepared and topped with Whatman #10 filter paper before the CMA set. 1000 μL of a conidial suspension (1 × 10^6^ spores mL^−1^) of *L. uredinophilum* transformant PP2018‐001‐GFP_08 was pipetted onto the surface of the growth media and gently spread across the plate using a sterile glass rod and incubated at 25°C ± 2°C for 14 days. Approximately 1 mg of freshly stored (48 h at 4°C) urediniospores of ASR with a germination percentage of 80%–100% were dusted onto the 14‐day‐CMA‐filter‐paper cultures of PP2018‐001‐GFP_08 with the aid of a small paint brush and incubated at 25°C ± 2°C for 5 days. Each day of the 5 days, urediniospores were picked onto a glass slide, covered with a cover slip, and edges fixed onto the glass slides with nail wax polish. The slides were immediately observed using a confocal microscope (Zeiss LSM 710 Confocal Laser Scanning Microscope, Zeiss, Oberkochen, Germany) equipped with a Zeiss camera and Zeiss Zen imaging software for image acquisition and processing, respectively. Confocal images were generated with a 63 × 1.4 NA Apo lens and in conjunction with a 488 nm krypton/argon laser. The laser was able to excite the GFP in *L. uredinophilum* (hyphae and conidia), as well as autofluorescent compounds in *P. pachyrhizi* urediniospores. The PP2018‐001‐GFP_08 image acquisition spectra were 510–535 nm, whereas urediniospores' autofluorescence spectra were 550–650 nm.

### 2.8 Transmission Electron Microscopy (TEM)

The TEM experiment followed the protocol of Gauthier et al. (2014) with some slight modifications (formalin‐acetic acid‐alcohol, FAA was not used). Transmission electron microscopy samples were highly dependent on SEM output to limit the number of samples to less than ten of the 23 SEM samples (to maximize the time of the elaborate TEM processes and limit unnecessary duplications). Stored samples (both leaf and the CMA‐filter‐paper cultures) in the primary fixation phase (3% glutaraldehyde in 0.05 M in sodium cacodylate buffer) were retrieved and washed in 0.05 M sodium cacodylate buffer four times at 30‐min intervals and rinsed in the same, but fresh, buffer for 24 h in vials, with the buffer completely covering the submerged samples. After removal of the overnight buffer, osmium tetroxide (2%) in 0.05 M sodium cacodylate buffer was used to post‐fix the samples for a duration of 3 h (or until the samples turned black), followed by rinsing the samples in 0.05 M cacodylate buffer twice at 30‐min intervals. A graded series of ethanol dehydration of samples from 10% to 100% at 10‐min washing periods was maintained up to the 90% wash and culminated in two 10‐min washes in 100% ethanol. After dehydration, samples were placed in 100% propylene oxide for two 15‐min intervals. An infiltration process using LR white resin (polyhydroxylated acromatic acrylic resin) was followed, using a graded series at 1 h intervals (25%, 50%, 75%, 100% resin mixed with 75%, 50%, 25% and 0% of propylene oxide). Samples were left overnight in 100% resin. Molds for samples were filled up to 50% with 100% resin, and each sample was placed in the mold and topped up with 100% resin to create a convex shape. Embedded samples in the molds were polymerized in the oven at 70°C overnight (16–24 h), removed from the oven to cool, the blocks removed from the molds and were subsequently labeled accordingly. Glass knives for sectioning were cut using an LKB 780 1A Knifemaker (Stockholm‐Bromma, Sweden). After blocks were acquired, they were rough trimmed and thin sectioned (at 500 nm) with a Reichert‐Jung Ultracut E Ultramicrotome (DiATOME, USA) and ultra‐thin sectioned (at 120 nm) with a Leica UC7 Ultramicrotome (Wetzlar, Germany). Thin sections were picked up and dried onto glass slides using a hot plate and stained with toluidine blue. Ultrathin sections were picked up onto copper grids and post‐stained with 2% uranyl acetate (5 min) and lead citrate (5 min) with a 30‐s wash in deionized water in between. Images were acquired with Gatan's Digital Micrograph (DMG) software using a Gatan Orius SC‐600 CCD camera (AMETEK Inc., USA) in the TEM (JEM‐1400, JEOL, Tokyo, Japan).

## 3 Results

### 3.1 Scanning Electron Microscopy (SEM)

**FIGURE 3:**
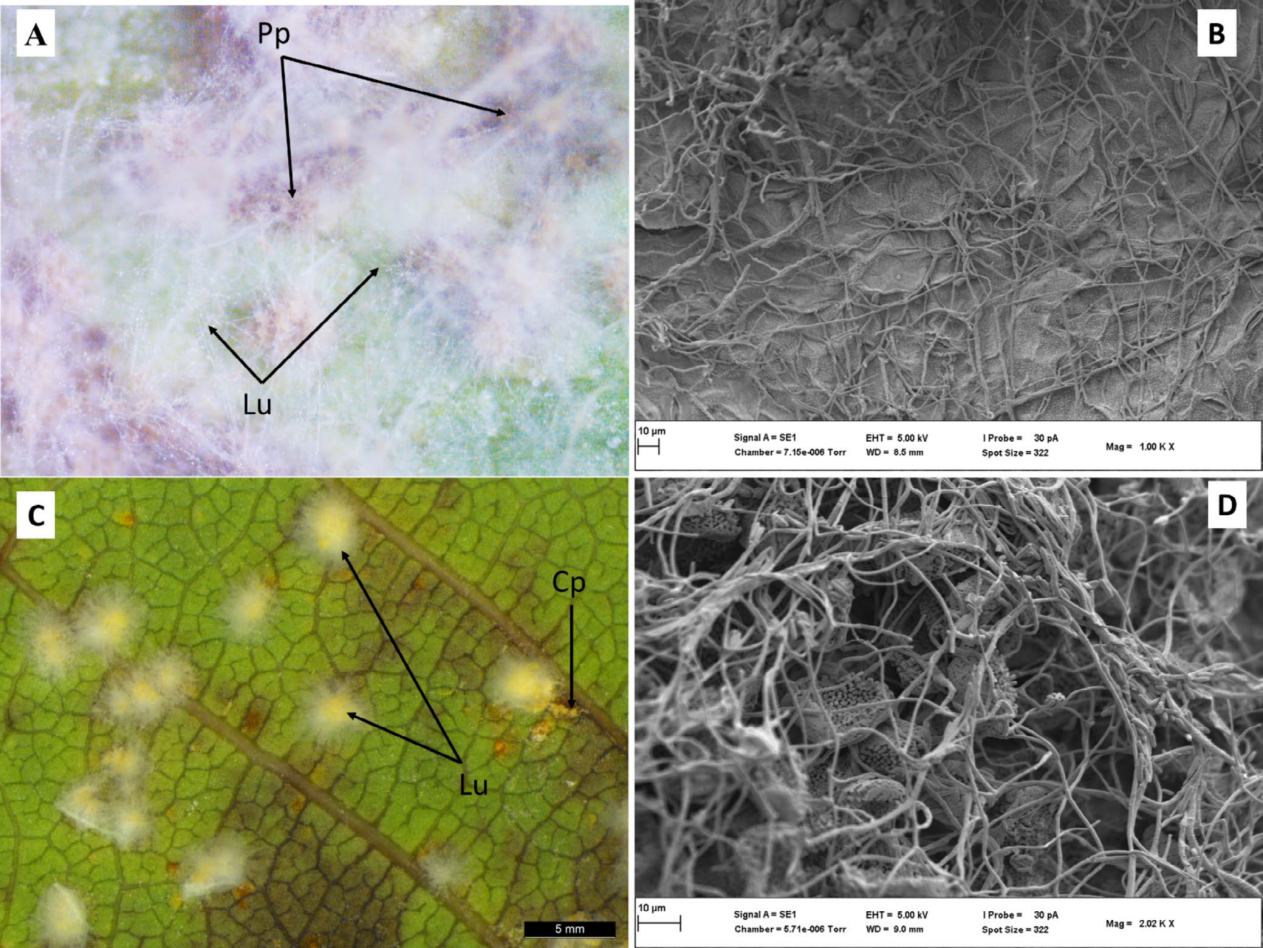
Light and SEM views of hyperparasitism by *L. uredinophilum*: stereomicroscope views of a soybean leaf cutting infect by *P. pachyrhizi* (Pp) rust pustules (brown in color) colonized by *L. uredinophilum* (Lu) white mycelia (A); scanning electron micrograph showing a network of *L. uredinophilum* on a soybean leaf surface (B); a frangipani (*Plumeria* spp.) leaf cutting infected by *C. plumeriae* (Cp) forming pustules, which are colonized by *L. uredinophilum* (Lu), 5 dpi (C); Scanning micrograph showing *L. uredinophilum* on colonized pustules of *C. plumeriae* (D).

**FIGURE 4:**
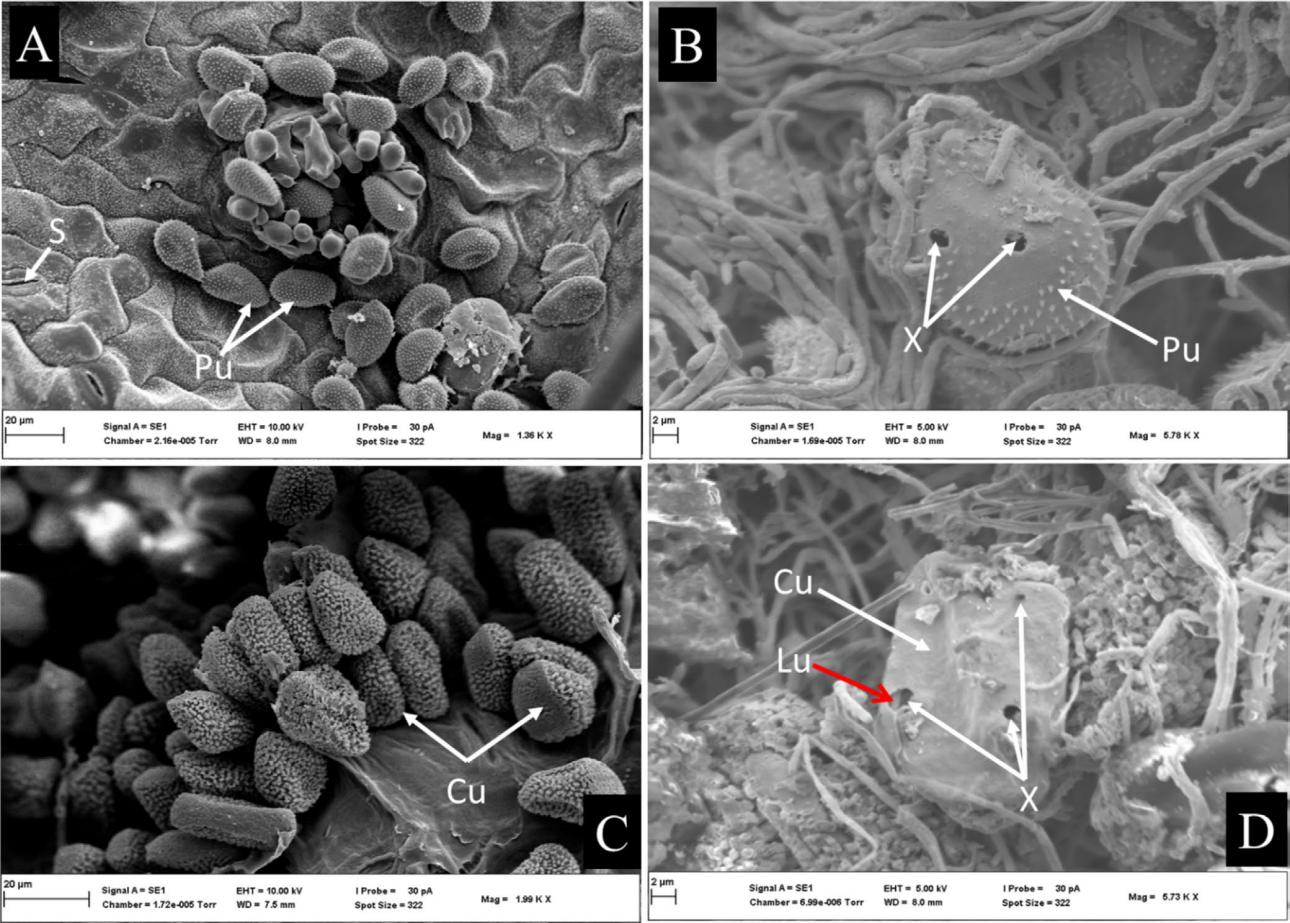
Scanning electron microscope view *Phakopsora pachyrhizi* urediniospores (Pu) not treated with *L. uredinophilum* spore suspension (A); *Phakopsora pachyrhizi* urediniospores (Pu) with perforations (X) on them (B); urediniospores of *C. plumeriae* (Cu) not treated with *L. uredinophilum* suspension spores, (C); a perforated urediniospore of *C. plumeriae* (Cu) and the red arrow shows a hypha of *L. uredinophilum* (Lu) growing inside the urediniospore of *C. plumeriae* (D).

**FIGURE 5:**
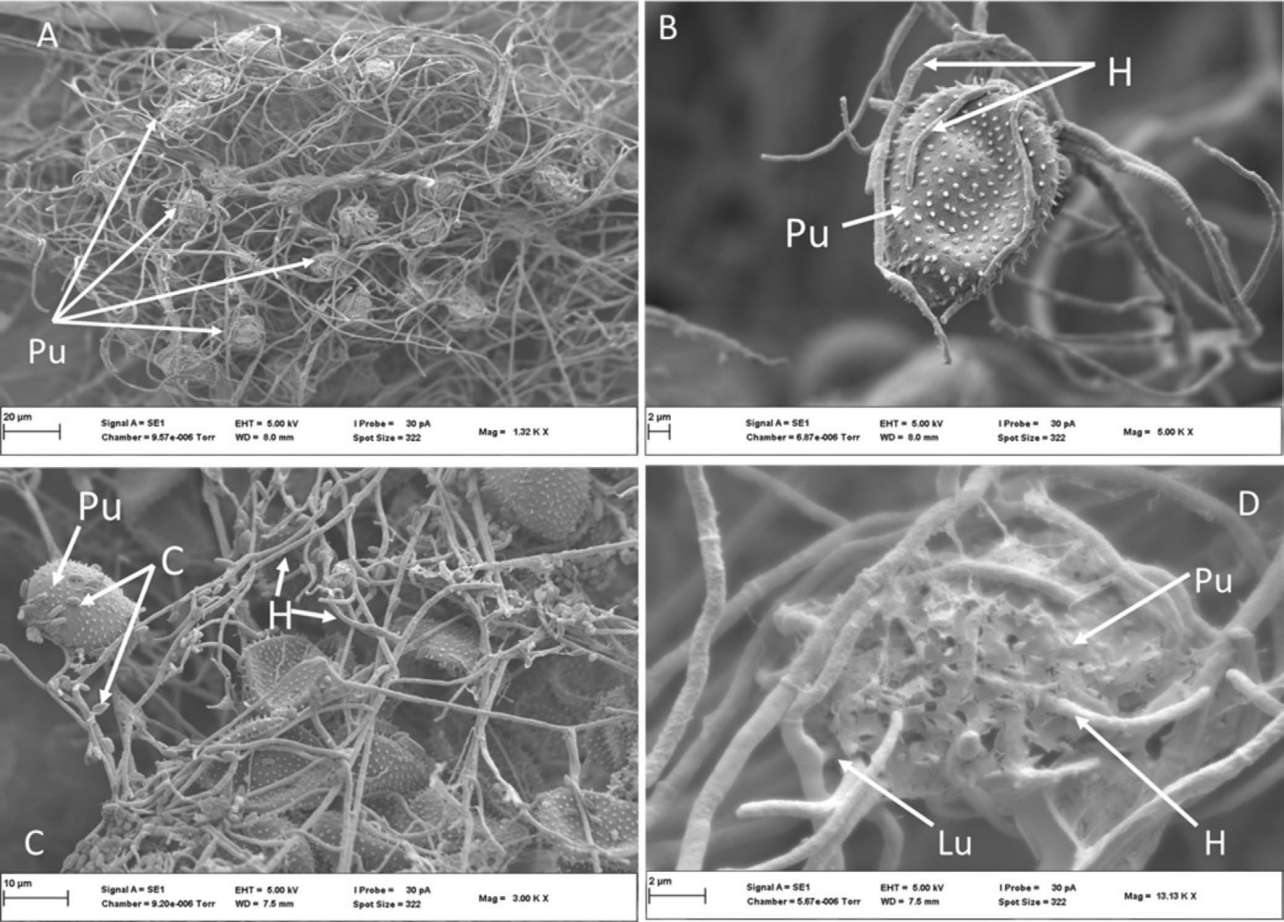
Scanning electron microscope view of the interactions of *L. uredinophilum* and *P. pachyrhizi*: an extensive hyphal network of *L. uredinophilum*, with some hyphae tightly coiled around urediniospores of *P. pachyrhizi* (Pu) 3 dpi (A); an individual urediniospore of *P. pachyrhizi* (Pu) being colonized by L. uredinophilum hyphae, 3 dpi (B); Conidia (C) on the surface of urediniospores (Pu), as well as a network of hyphae (H), (C); a urediniospore (Pu), with a disintegrating cell wall, with penetration by hyphae (H) of *L. uredinophilum* (Lu), 5 dpi (D).

**FIGURE 6:**
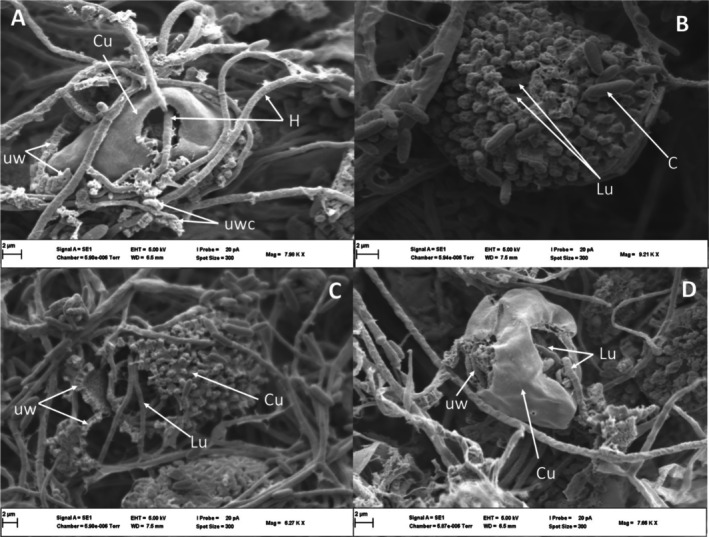
Scanning electron microscope views of interactions of *L. uredinophilum* and *C. plumeriae*: a urediniospore of *C. plumeriae* (Cu) showing a substantial hole, with hyphae of *L. uredinophilum* penetrating through the perforation, as well as breakdown of the outer membrane of the urediniospore, and cell wall damage (urediniospore wall component—uwc) (A); Conidia and mycelia growing inside and outside a urediniospore, after the outer membrane of the urediniospore had been compromised (B); breakdown of the urediniospore cell wall (uw), 5 dpi (C); extensive damage to a *C. plumeriae* urediniospore (Cu), showing a damaged cell wall (uw), and hyphae of *L. uredinophilum* (Lu)., 10 dpi (D).

**FIGURE 7:**
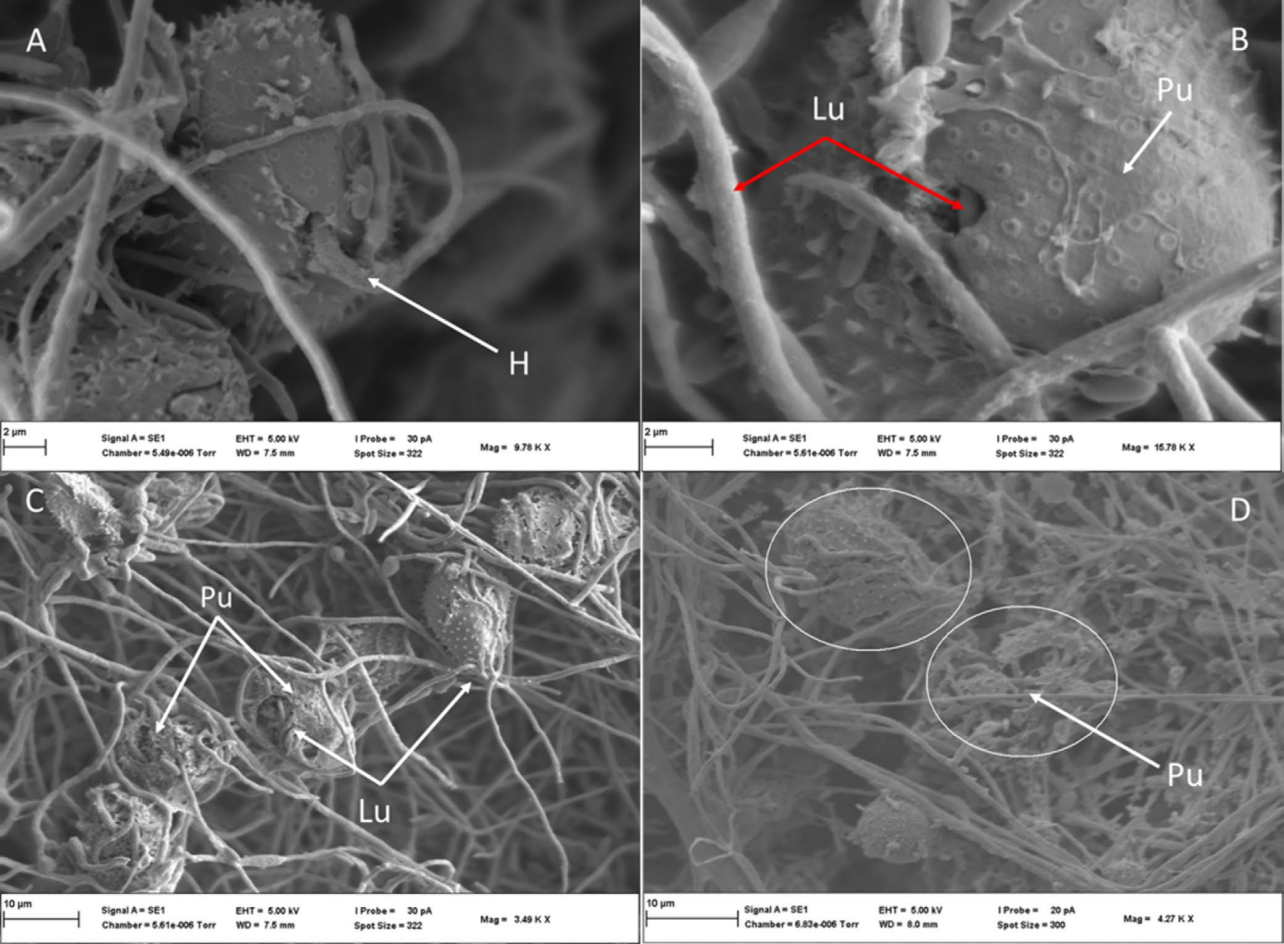
Scanning electron microscope view hyperparasitism of urediniospores of *P. pachyrhizi*: a urediniospore being penetrated by a hypha (H) of *L. uredinophilum* (A); a hypha of *L. uredinophilum* growing inside a urediniospore, viewed through a perforation (B); several urediniospores (Pu) being parasitised by hyphae of *L. uredinophilum* (Lu), resulting in damaged cell walls (C); interactions in a filter‐paper‐culture, 10 dpi, several urediniospores (Pu) can be seen to have started disintegrating (D).

Soybean leaf surfaces were not penetrated by *L. uredinophilum*; however, hyphae could be seen extending towards where there were urediniospores (Figure 3B) and in vivo inoculations did result in the colonization of rust pustules (Figure 3A for *P. pachyrhizi* and Figure 3C for C. *plumeriae*). At 7 days post inoculation (dpi), pustules of *P. pachyrhizi* and *C. plumeriae* showed clear perforations on urediniospores of both *P. pachyrhizi* (Figure 4B) and *C. plumeriae* (Figure 4D) as compared to the control (Figure 4A, *P. pachyrhizi*) and (Figure 4C, *C. plumeriae*). Moreover, the integrity of the urediniospores for both rust species appeared compromised (compared to the control). Some mycelia could be seen growing inside the urediniospore of *C. plumeriae* (Figure 4D). The PP2018‐001‐GFP filter paper cultures showed urediniospores extensively coiled in a mat of *L. uredinophilum* hyphae (Figure 5A). It showed microbe‐microbe interactions and a concentration of growth around the urediniospores after 3 days on the filter‐paper culture. Individual urediniospores could be seen under attack by *L. uredinophilum* (Figure 5B), suggesting some signaling that draws the mycoparasite to the urediniospores. An extensive hyphae network and conidia on the surface of the urediniospores could also be seen (Figure 5C). The *L*. *uredinophilum* hyphae could be seen protruding out, and some ramifying through the *P. pachyrhizi* urediniospore (Figure 5D). The CMA‐filter‐paper culture co‐inoculations with *C. plumeriae* at 5 dpi were able to show *L. uredinophilum* hyphae growing through perforations of urediniospores. Mycelia growing inside the urediniospores were clearly visible after their gritty outer layers were compromised or peeled off (Figure 6A,B). Moreover, the *C. plumeriae* urediniospore could be seen disintegrating with *L. uredinophilum* mycelia ramifying through the disintegrating urediniospore (Figure 6C) and more perforations and disintegration were observed at 10 dpi (Figure 6D). Soybean rust urediniospores also did show clear penetration by *L. uredinophilum* (Figure 7A). Mycelia of *L. uredinophilum* growing inside the urediniospore were viewed through a pore on the urediniospore (Figure 7B). A series of visibly damaged urediniospores and loss of urediniospore membrane wall could be seen (Figure 7C), as well as after 10 dpi, the outer membrane and urediniospores could be seen with clear disintegration of the urediniospores (Figure 7D).

### 3.2 Confocal‐Laser Scanning Microscopy (CLSM)

**FIGURE 8:**
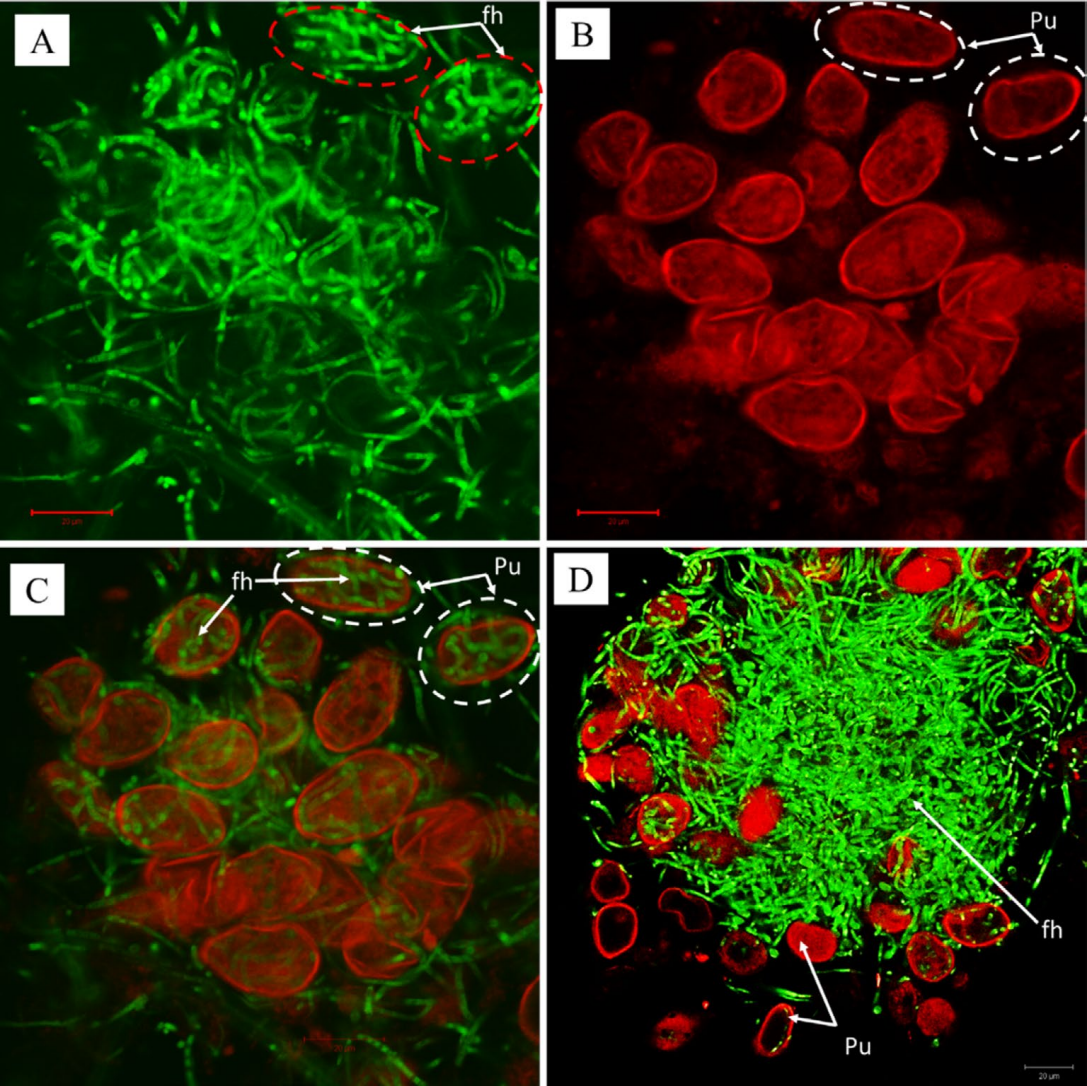
Confocal laser‐scanning microscope views: green fluorescence shown by a GFP—*L uredinophilum* transformant at the 510–535 nm emission spectra; circled in red visible hyphae (A); *Phakopsora pachyrhizi* urediniospores (Pu), non‐circled and circled in white, obtained at 550–650 nm emission spectra (B); circled in white are urediniospores with the green fluorescent GFP‐*L. uredinophilum* hyphae inside the urediniospores (C); a large mass of fluorescing GFP‐*L. uredinophilum* hyphae (fh) with several of these hyphae appearing to be inside or on the surface of *P. pachyrhizi* urediniospores (D).

**FIGURE 9:**
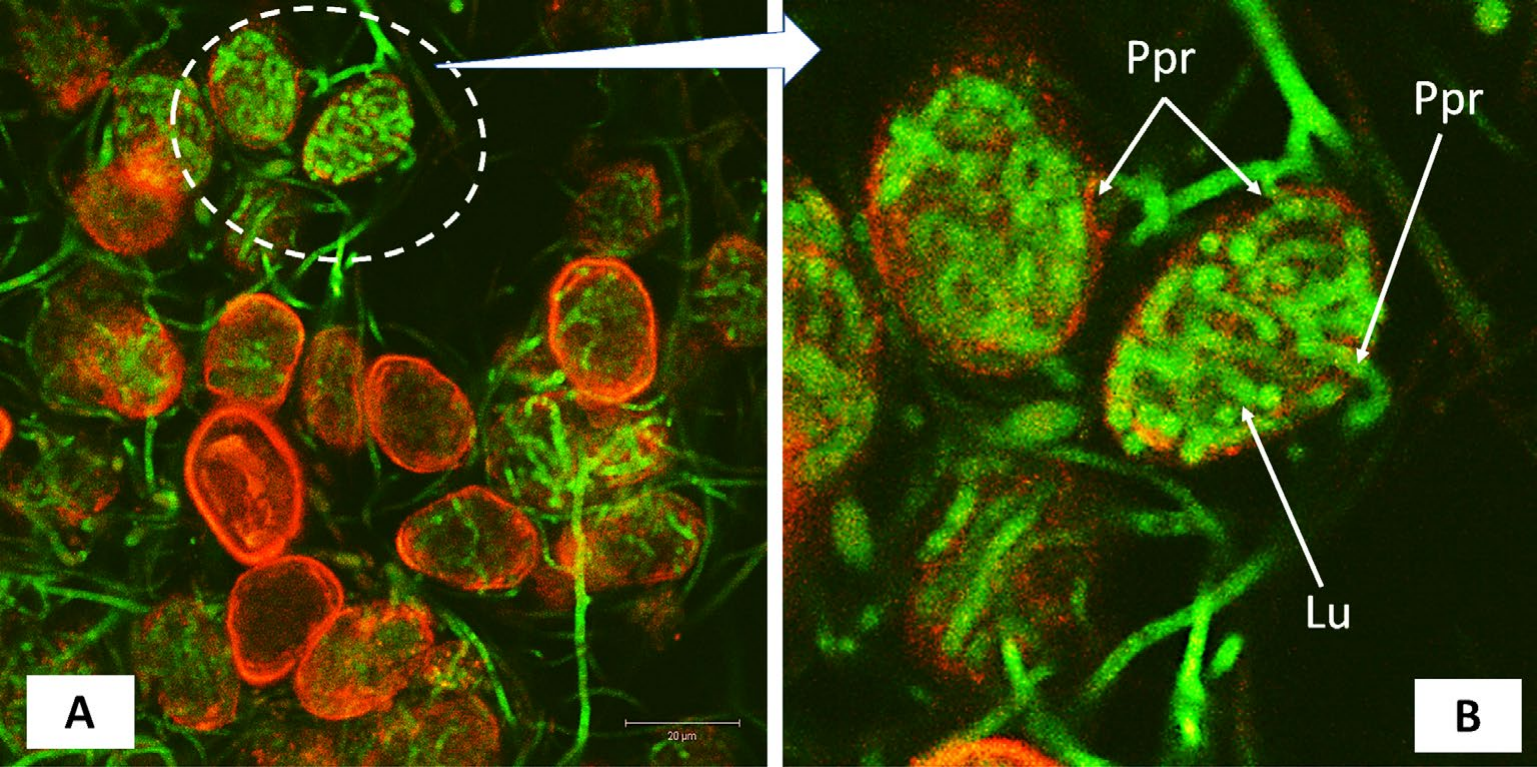
Confocal laser‐scanning microscope views: an extensively colonized urediniospores of *P. pachyrhizi* by *L. uredinophilum* (Lu), 72 h after co‐inoculations on a CMA‐filter‐paper‐culture (A); an enlarged image of the same site, showing possible points of penetration (Ppr), or exit, of hyphae from the urediniospore by *L. uredinophilum* (B).

The PP2018‐001‐GFP_08 (*L. uredinophilum* transformant with GFP) colonized *P. pachyrhizi* urediniospores leaf and CMA‐filter‐paper cultures. After 76 h post‐co‐culture, at least 60% to 80% of urediniospores were colonized and showed fluorescence due to the GFP. They showed extensive growth inside urediniospores (Figure 8A,C). The soybean rust urediniospores auto‐fluoresced red (514 nm wavelength), whilst the PP2018‐001‐GFP_08 showed fluorescence at a wavelength of 488 nm. The CLSM revealed possible penetration sites through either germ tube pores of urediniospores and possible direct penetration (Figure 9B), and the *L. uredinophilum* hyphae were extensively and tightly coiled inside the urediniospores.

### 3.3 Transmission Electron Microscopy (TEM)

**FIGURE 10:**
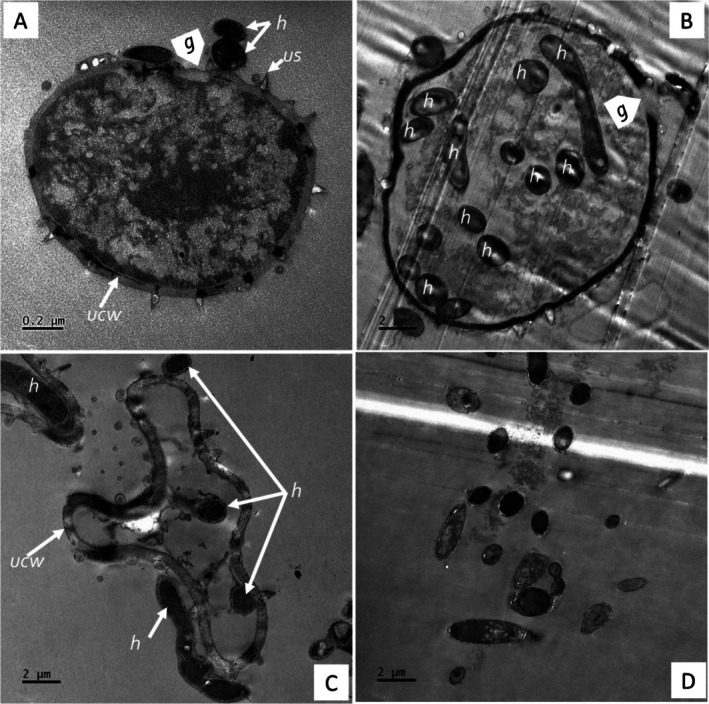
Transmission electron microscope view of the soybean rust urediniospores (*Phakopsora pachyrhizi*) interacting with the hyperparasite (*L. uredinophilum*) on CMA‐filter‐paper‐culture 24 to 36 h post‐co‐inoculations: a urediniospore with a visible germ pore point (arrowhead) (*g*) before colonization by the hyperparasite (A). Note the urediniospore spine (us), urediniospores cell wall (ucw) and hyphae (*h*) of the hyperparasite growing outside the urediniospore of soybean rust; a germ tube point of infection (*g*) and numerous hyphae (*h*) inside the urediniospore (B); a distorted urediniospore cell wall (cw) shape, with hyphae (*h*) inside the urediniospore (C); a cross‐section of conidia of *L. uredinophilum* (D).

**FIGURE 11:**
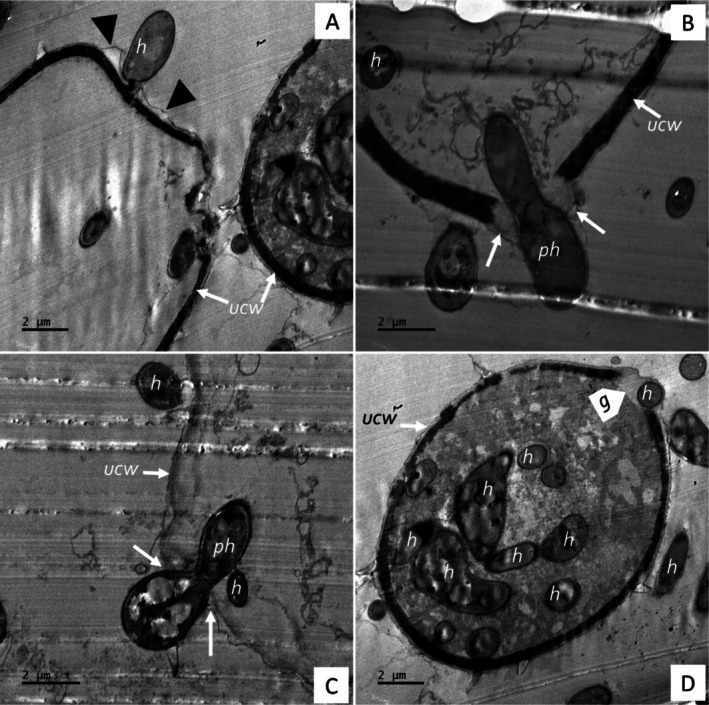
Transmission electron microscope view of the soybean rust urediniospores (*P. pachyrhizi*) interacting with the hyperparasite (*L. uredinophilum*) on CMA‐filter‐paper‐culture 24–36 h post‐co‐inoculations: a conidium of *L. uredinophilum* in direct contact with a urediniospore, with mucilaginous matrix (black arrowheads), probably gluing the conidium to the urediniospore (A); penetration by a hypha‐penetrating hypha (ph) of *L. uredinophilum* into a urediniospore, showing degradation of germ pores (*g*) indicated by arrows, or with a matrix (arrows) on the outside of the hyphae at the point of penetration (B); another view of a hypha of *L. uredinophilum* penetrating a urediniospore, with a matrix (arrows) on the outside of the hyphae at the point of penetration (C); hyphae (*h*) growing inside a urediniospore (D).

**FIGURE 12:**
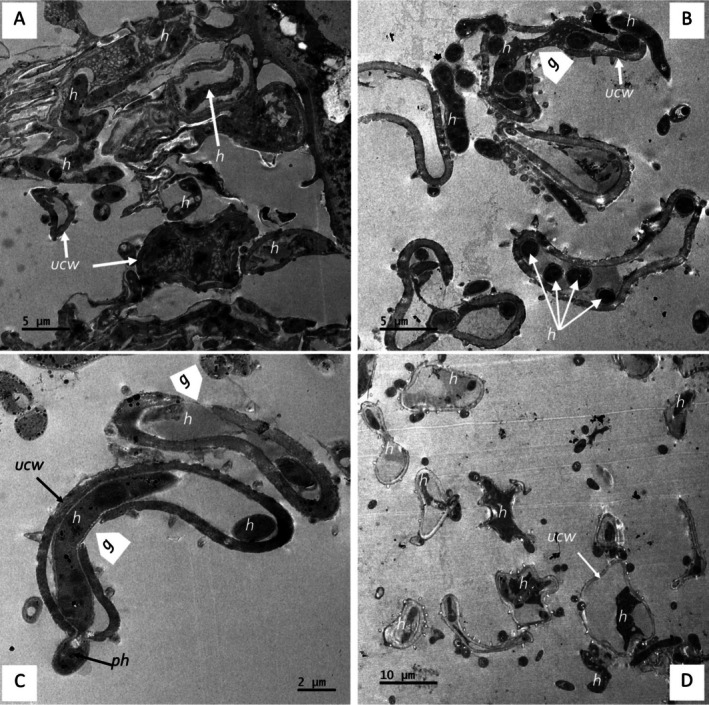
Transmission electron microscope view of the soybean rust urediniospores (*P. pachyrhizi*) interacting with the hyperparasite (*L. uredinophilum*) in vivo, 5 days post spraying with hyperparasite spores: a burst soybean rust pustule showing infected urediniospores and the hyphae (*h*) of *L. uredinophilum* (A); urediniospores colonized by hyphae (*h*) of *L. uredinophilum*, the cell walls of the urediniospores remain intact but distorted (B); a hypha (ph) of *L. uredinophilum* penetrating a urediniospore and expanding inside. The cell wall (uwc) remains intact, and a germ pore can be seen (*g*) (C); hyphal growth in several soybean rust urediniospores collapsed urediniospores due to hyperparasitism (C). Urediniospores germ pores (*g*); urediniospores cell wall (ucw) hyphae (*h*) of the hyperparasite growing inside the urediniospore of soybean rust, penetrating hypha (ph) (D).

**FIGURE 13:**
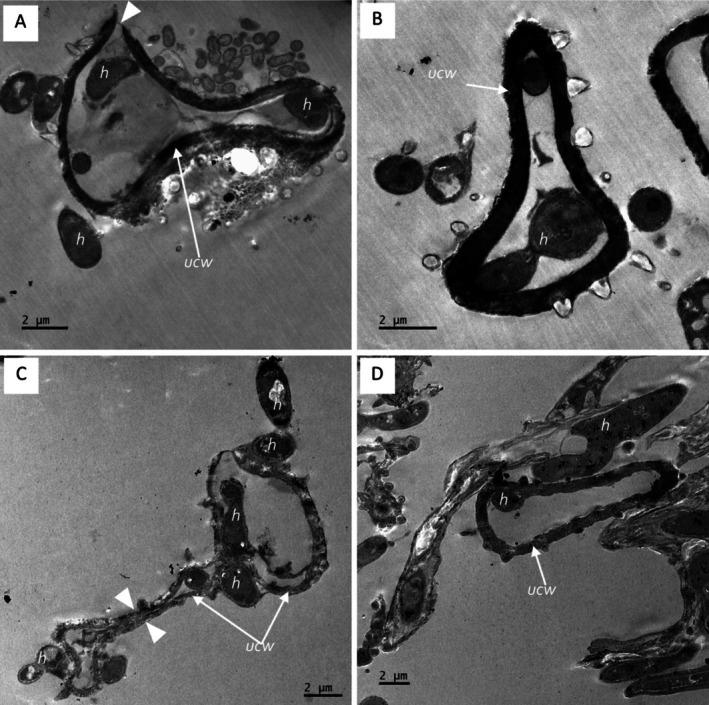
Transmission electron microscope view of the soybean rust urediniospores (*P. pachyrhizi*) interacting with the hyperparasite (*L. uredinophilum*) in vivo, 10 days post spraying with hyperparasite spores: a urediniospore with a broken cell wall (uwc) (arrowhead) and hyperparasite colonization, with hyphae inside (*h*) (A); advanced hyperparasite hyphal growth (*h*) in a urediniospore. The cell wall (uwc) remains intact (B); two collapsed urediniospore, with their cell walls still intact (uwc) (white arrows), and hyphae (*h*) of growing inside an adjacent urediniospore (C); a urediniospore in a burst pustule, colonized by *L. uredinophilum* hyphae (h) (D).

The ultra‐thin TEM section for the co‐inoculations in the CMA‐filter‐paper‐culture in vitro studies (24–36 h) revealed the soybean urediniospores' cellular integrity (although organelles were not distinctive), as shown in Figure 10A, germ pore sites (Figure 10A,B). Urediniospores were internally colonized by growing hyphae of the hyperparasite (Figure 10B). Some urediniospores were visibly distorted in shape, possibly due to infection by the hyperparasite (Figure 10C). In contrast, the control did not show any visible distortions. The hyperparasite *L. uredinophilum* hyphae and spores (Figure 10D) were visible around the urediniospores. No urediniospore germination was observed in the in vitro studies. Further observation on the infection sites on urediniospores indicated possibly three strategies prior to infection: (i) the hyperparasite spores or hyphae attached themselves to the urediniospores through somewhat mucilaginous matrix (Figure 11A); (ii) infection through penetrating urediniospores via germ tube degradation as observed in Figure 11B; and (iii) direct penetration through mechanical force (Figure 11C). After these infection strategies, the hyperparasite showed putative hyphal growth inside urediniospores leaving clear germ pore entry sites (Figure 11D). The in vivo studies (5–10 days) revealed some burst soybean rust pustules with some urediniospores colonized by the hyperparasite *L. uredinophilum* hyphae (Figure 12A,B,D). Urediniospores of the soybean rust lost cellular integrity and shape and some visible germ pore sites as well as direct penetrating hyperparasite *L. uredinophilum* hypha and hyphal growth into the cell or out of the urediniospores (Figure 12C,D). The cytoplasm of the colonized urediniospore varied in appearance, some not clearly showing contents, or some became vacuolated with clear distortions of cellular contents or emptying of contents following successful infection by the hyperparasite. Further in vivo results revealed putative hyphal growth of *L. uredinophilum* (Figure 13A–C), a break in a cell wall of a urediniospore (Figure 13A). A collapse and distortion in the shape of urediniospores confronted by *L. uredinophilum* was also observed (Figure 13C) whilst in a burst pustule, a urediniospore colonized by the hyperparasite was observed (Figure 13D).

## 4 Discussion

Fungal parasitism has been evaluated and targeted as a potential biological control strategy for phytopathogens, for example, effective control of mildew on grapes and other crops by *Ampelomyces quisqualis* (Menge and Makobe 2017; Németh et al. 2023; Saha et al. 2021; Sztejnberg 1993). The current study was able to document the fungus‐fungus parasitism of *L. uredinophilum* isolate PP2018‐001 on *P. pachyrhizi* both in vivo and in vitro, and such antagonist interactions as have been observed are well supported by definition as mycoparasitism (Barnett 1964; Karlsson et al. 2017; Mukherjee et al. 2022; Van Den Boogert 1996; Wang et al. 2023; Zeilinger‐Migsich and Mukherjee 2014). The fungi genus *Lecanicillium* spp. (formerly *Verticillium* for some species) is well known as hyperparasites forming fungus‐fungus antagonist interactions with lesions of various fungal diseases of plants, such as rusts (Park et al. 2015; Setiawati et al. 2021; Wei et al. 2018), powdery mildews (Askary et al. 1997; Kim et al. 2007, 2010; Miller et al. 2004), leaf spots (Nana et al. 2022) and others reviewed by (Goettel et al. 2008). The hyperparasite *L. uredinophilum* could infect *P*. *pachyrhizi* urediniospores within 24 h, making their contents a nutritive substrate and rendering the parasitized urediniospores non‐viable. Through mycoparasitic interactions, the *L. uredinophilum* hyphae tightly coiled around urediniospores, directly penetrated urediniospore cell walls, and degraded germ pore sites to gain access into and exit from the *P. pachyrhizi* urediniospores. Similar observations were recorded for parasitized rust urediniospores of *C. plumeriae*. There are documented studies of rust pathogens parasitized by other fungi. For example, the coffee rust fungus (*Hemileia vastatrix* Berkeley & Broome) has been reported to be hyperparasitized by the entomopathogenic fungus *Cordyceps cateniannulata* Z.Q. Liang, through mycoparasitism. This fungus also acted as an endophyte and entomopathogenic to *Tenebrio molitor* (mealworms), *Hypothenemus hwamei* (coffee berry borer beetle) and a leaf miner (*Leucoptera coffeella*) (Pereira et al. 2024). Other documented examples include *Akanthomyces lecanii* on peanut rust (*Puccinia arachidis* Speg.) (Nana et al. 2023, 2024), *Hemileia vastatrix* coffee rust hyperparasitized by *Lecanicillium lecanii* (Belachew Bekele 2022; Das et al. 2024; Jackson et al. 2012; Vandermeer et al. 2009), *Verticillium psalliotae* Treschow on soybean rust (*P*. *pachyrhizi*) (Saksirirat and Hoppe 1990, 1991) and *Puccinia* spp. hyperparasitized by *Cladosporium uredinicola* (Barros et al. 1999; Traquair et al. 1984).

The CLSM investigations allowed the visualization of the *L. uredinophilum* GFP transformant, putative and near‐complete internal colonization of *P. pachyrhizi* urediniospores, and entry or exit sites within 36–96 h from PDA‐filter‐paper‐culture co‐inoculations, similar to Gauthier et al. (2014) in their study using *Simplicillium lanosoniveum* GFP transformant as their hyperparasite on *P. pachyrhizi* urediniospores. The same authors also observed extensive branching of hyperparasite hyphae upon entry into *P. pachyrhizi* urediniospores and over 90% colonization on day 5, similar to the current study. The rapid hyperparasitic infection of *P. pachyrhizi* urediniospores is crucial in that it has the potential to drastically reduce the secondary inoculum of the highly polycyclic and sporulating ASR fungi and, therefore, curtail the rate at which ASR epidemics can occur. Gauthier et al. (2014) successfully revealed this strategy in their field studies with *S. lanosoniveum* against ASR.

*Lecanicillium uredinophilum*, through mycoparasitism, formed appressorium‐like structures in confrontation and infection of the urediniospores. The infection led to the formation of penetration structures similar to those observed by (Nana et al. 2023) in their studies of *Akanthomyces lecanii* against *Puccinia arachidis* (peanut rust) and similarly observed attachment to urediniospores through the mucilaginous matrix, which has been suggested to create a conducive environment for enzymatic activity and insect cuticle penetration by *V. lecanii* (Schreiter et al. 1994). Other previous studies, such as mycoparasitism of *Uromyces appendiculatus* (bean rust) by *Cladosporium tenuissimum*, revealed a possible attack of urediniospores by mechanical force and suggested enzymatic activity (Assante et al. 2004). Gauthier et al. (2014) recorded similar results when *S. lanosoniveum* parasitised urediniospores of *P. pachyrhizi*, and some of their observations suggested possible enzymatic activity. This study also suggests enzymatic activities in the infection process due to the presence of the mucilaginous matrix, which aids in the attachment of the hyperparasite to the host and, in the process, releases lytic enzymes.

Furthermore, the current study reveals, through the SEM, the disintegration or peeling of the outer urediniospore walls and detachment of spines from the urediniospores. This act is phenomenal for enzymatic activity. Early studies, for example, by Saksirirat and Hoppe (1991), were able to reveal that *V. psalliotae* and *V. lecanii* secreted a battery of enzymes (glucanases, chitinases, proteases) upon culture with autoclaved *P. pachyrhizi* urediniospores. *Verticillium lecanii* was reported to produce amylases, lipases, and proteases in another study by (Hasan et al. 2013), whereas Askary et al. (1997), through their cytochemical investigation on *V. lecanii* against *Sphaerotheca fuliginea* (Schlechtendahl) U. Braun & S. Takamatsu (cucumber powdery mildew), implicated the release of cell‐wall degrading enzymes (CWDEs) after attachment to their fungal host. *Lecanicillium attenuatum* applied to rice fields to control the brown planthopper ( *Nilaparvata lugens* [Stål]) upon transcriptome sequencing analysis showed high gene expression in the production of four principal enzymes (proteases, phospholipases, cutinases, chitinases), which are responsible for the digestion of the insect tissue (Zhang et al. 2024).

Transmission scanning microscopy is endowed with the ability to resolve microbe‐microbe interactions, such as those of *L. uredinophilum* versus *P. pachyrhizi*, as well as microbe‐plant interactions, *P. pachyrhizi* versus the soybean plant at the cellular level. Microbe‐microbe interactions in biological control are essential in elucidating the mechanism of the infection process, which the TEM study corroborates well with other microscopy work (SEM and CLSM). The TEM experiment investigated whether *L. uredinophilum* gained access into the urediniospores via direct urediniospore wall penetration, germ tube pores, and evidence or lack thereof of degraded urediniospore outer membranes before colonization and infection. The TEM also sought to examine subcellular membranes post‐*L. uredinophilum* urediniospores hyperparasitism.

Several studies investigating mycoparasitism (including the current study) have shown quite striking similarities, such as degradation of urediniospore germ pores, extensive hyphae growth inside the host fungi urediniospores, disintegration of urediniospore contents, and loss of cellular integrity (Askary et al. 1997; Gauthier et al. 2014; Nana et al. 2023; Spencer 1980; Spencer and Atkey 1981). The SEM investigations revealed that the outer walls of urediniospores peeled off and spines were removed, which could be due to enzymatic activity or mechanical penetration by the *L. uredinophilum* hyperparasitism; the TEM did not reveal degradation or lack of urediniospore cell walls for most observed cells, although some images suggested partial digestion. Urediniospore exhibited cell wall breaks and multiple germ tube sites. In contrast, another TEM study by Mendgen (1981) observed partial and dissolved urediniospore walls upon confrontation of stripe rust (*Puccinia striiformis*) with *V. lecanii*. Despite these deviations, the current research extensively elucidates the attack of *P. pachyrhizi* urediniospores by *L. uredinophilum*, which gains nutrition through this characteristic interaction and a convincing act of mycoparasitism (Assante et al. 2004; Gauthier et al. 2014; Gohel et al. 2022; Nana et al. 2023).

In conclusion, this current study demonstrated that *L. uredinophilum* employs mycoparasitism as one of its primary strategies for effective infection and killing *P. pachyrhizi* urediniospores. Through high‐end microscopy (CLSM, SEM and TEM) for treatments done for the CMA‐filter‐culture co‐inoculations and on soybean rust‐infected living host, a summary of at least four major events can be deduced through which *L. uredinophilum* acts as a hyperparasite and mycoparasite of *P. pachyrhizi*. The four events observed in the current study are (i) attachment of the hyperparasite *L. uredinophilum* to host fungi urediniospores (coiling of hyphae around urediniospores and spore attachment to urediniospores); (ii) mechanical force through direct penetration of urediniospore walls by *L. uredinophilum* or clear entry through degradation of germ pores (suggestive of enzymatic activity); (iii) penetration and active growth of the hyperparasite *L. uredinophilum* hyphae inside urediniospores; and (iv) digestion of urediniospore contents by *L. uredinophilum* leading to collapse and contortion of urediniospores and release of hyperparasite from dead urediniospores. Therefore, the isolation of *L. uredinophilum* from wild strawberry rust was not a coincidence. It strongly suggests, and has been investigated, that it can use rusts as a substrate for its nutrition through mycoparasitism, which gives rise to its potential use in the biological control of the ASR pathogen. Molecular fungus‐fungus interaction could be studied to elucidate the mechanism of action, including how genes influence the establishment of successful interactions. Application studies of *L. uredinophilum* could focus on the controlled environmental conditions required for effective control of the ASR pathogen and focus on producing formulations that can be used for field trial applications.

## Disclosure

All the authors of the manuscript titled “Ultrastructural Examination of the Fungus‐To‐Fungus Interactions of *Lecanicillium uredinophilum* and *Phakopsora pachyrhizi*,” agree to its submission to Plant‐Environment Interactions.

## Ethics Statement

This article does not contain any studies with human participants or animals conducted by any of the authors.

## Conflicts of Interest

The authors declare no conflicts of interest.

## Data Availability

Data sharing does not apply to this article, as all relevant images generated during the study are fully presented and described within the article.
